# The acute effects of higher versus lower load duration and intensity on morphological and mechanical properties of the healthy Achilles tendon: a randomized crossover trial

**DOI:** 10.1242/jeb.243741

**Published:** 2022-05-13

**Authors:** Eman Y. Merza, Stephen J. Pearson, Glen A. Lichtwark, Peter Malliaras

**Affiliations:** 1 Department of Physiotherapy, Faculty of Medicine, Nursing and Health Science, Monash University, Frankston, VIC 3199, Australia; 2 Centre for Health, Sport and Rehabilitation Sciences Research, University of Salford, Greater Manchester M5 4WT, UK; 3 Centre for Sensorimotor Performance, School of Human Movement and Nutrition Sciences, The University of Queensland, St Lucia, QLD 4072, Australia

**Keywords:** Achilles tendon, Volume, Stiffness, Free tendon, 3D ultrasound

## Abstract

The Achilles tendon (AT) exhibits volume changes related to fluid flow under acute load which may be linked to changes in stiffness. Fluid flow provides a mechanical signal for cellular activity and may be one mechanism that facilitates tendon adaptation. This study aimed to investigate whether isometric intervention involving a high level of load duration and intensity could maximize the immediate reduction in AT volume and stiffness compared with interventions involving a lower level of load duration and intensity. Sixteen healthy participants (12 males, 4 females; age 24.4±9.4 years, body mass 70.9±16.1 kg, height 1.7±0.1 m) performed three isometric interventions of varying levels of load duration (2 s and 8 s) and intensity (35% and 75% maximal voluntary isometric contraction) over a 3 week period. Freehand 3D ultrasound was used to measure free AT volume (at rest) and length (at 35%, 55% and 75% of maximum plantarflexion force) pre- and post-interventions. The slope of the force–elongation curve over these force levels represented individual stiffness (N mm^−1^). Large reductions in free AT volume and stiffness resulted in response to long-duration high-intensity loading whilst less reduction was produced with a lower load intensity. In contrast, no change in free AT volume and a small increase in AT stiffness occurred with lower load duration. These findings suggest that the applied load on the AT must be heavy and sustained for a long duration to maximize immediate volume reduction, which might be an acute response that enables optimal long-term tendon adaptation via mechanotransduction pathways.

## Abstract (precis)

**Summary:** High levels of load duration and intensity have the greatest acute effect on the free Achilles tendon volume and stiffness.

## INTRODUCTION

Tendons are connective tissues composed of densely packed collagen fibres (solid phase) and water-binding proteoglycans (fluid phase) (Kjær, 2004). Tendons can adapt to a wide range of load requirements via changing their mechanical (i.e. stiffness) and morphological [i.e. thickness and cross-sectional area (CSA)] properties, and this may have implications for performance and injury (Bohm et al., 2015). For example, tendons become more extensible (Kubo et al., 2001b, 2009; Kay and Blazevich, 2009; Burgess et al., 2009; Joseph et al., 2014) and smaller in thickness (Wearing et al., 2007, 2014; Grigg et al., 2009; Kristiansen et al., 2014) when subjected to acute load, and become stiffer (Kubo et al., 2012, 2001a, 2006) and may hypertrophy (Arampatzis et al., 2007; Couppé et al., 2008; Bohm et al., 2014; Geremia et al., 2018; Kongsgaard et al., 2007) when loaded over long-term periods (>12 weeks). The mechanisms responsible for tendon adaptation to load are currently unclear.

It is well accepted that mechanical load raises hydrostatic pressure within the fluid phase, causing fluid flow (movement). This may include fluid exudation; that is, fluid movement out of the tendon causing reduced fluid volume, which has been demonstrated in *ex vivo* tendon loading (Butler et al., 1997; Lavagnino et al., 2003; Hannafin and Arnoczky, 1994). Tendons undergoing fluid flow/exudation may become thinner or smaller in volume. From a mechanical perspective, this means higher levels of mechanical stress on the solid phase (collagen) and a greater tissue strain. Moreover, load-induced fluid flow provides a mechanical signal for cellular activity (Docking et al., 2013; Wall et al., 2016). This is likely to occur because fluid flows away from the tendon core (it becomes thinner), so tensile tenocyte load may increase (as predicted by Poisson's ratio; Iwanuma et al., 2011). Shear stresses from the fluid flow may also directly impact tenocyte mechanotransduction and signalling (Archambault et al., 2002; Lavagnino and Arnoczky, 2005; Lavagnino et al., 2003, 2008). Therefore, it is reasonable to speculate that load-induced fluid flow/exudation is an important mechanism in tendon mechanotransduction and adaptation.

Fluctuation in fluid content (fluid loss) has been proposed as a potential mechanism for the reduction in tendon thickness/diameter observed post-acute loading (Wearing et al., 2007; Grigg et al., 2012, 2009). However, a reduction in tendon thickness could occur via fluid redistribution within the tendon with no exudation, and the latter may well change tendon morphology (volume) (Nuri et al., 2017c,a). The *in vivo* evidence on Achilles tendon (AT) volume change with acute loading is unclear. There are reports of a small but significant reduction in healthy AT volume immediately after cross-country running (Syha et al., 2014; Grosse et al., 2016), or a lack of acute change after eccentric (Obst et al., 2015) and submaximal isometric exercises (10 repetitions at 50% maximal voluntary isometric contraction, MVIC) (Nuri et al., 2017b), whilst others found an immediate reduction in tendinopathic AT volume with submaximal isometric exercises (at 50% MVIC) (Nuri et al., 2017c, 2018) or an immediate increase with eccentric calf training (Shalabi et al., 2004). Understanding load parameters that may trigger maximal reduction in tendon volume (meaning substantial fluid flow and exudation) may be useful in designing future loading protocols to drive the tendon toward optimal long-term adaptation (i.e. increased stiffness and tendon hypertrophy).

Volume changes of the AT are likely to be linked to the mechanical stress applied (e.g. load duration and intensity); however, there is a paucity of supporting evidence. It has been shown that tendons display greater deformation ‘creep’ when loaded slowly or for a longer duration because of their viscoelastic properties (Pearson et al., 2007) or when loaded heavily (i.e. >70% maximal voluntary contraction) (Obst et al., 2013). There is evidence that a combination of long-duration and high-intensity loading have the greatest acute and longer-term effects in healthy AT (Kubo et al., 2001c, 2005, 2009; Wiesinger et al., 2015; Obst et al., 2013). Kubo et al. (2009) compared short- (1 s, 5 sets×50 repetitions) and long-duration (15 s, 1 set×17 repetitions) high-intensity isometric contractions and found an immediate increase in AT elongation only after the long-duration contraction. Previous studies by the same group showed that long-duration and high-intensity isometric contractions over 12 weeks resulted in a greater increase in tendon stiffness compared with shorter-duration contractions (Kubo et al., 2001a, 2006). However, none of these studies linked changes in mechanical properties (i.e. stiffness) with changes in tendon morphology (i.e. volume). Whether and to what extent long-duration and high-intensity loading could lead to reductions in tendon volume and stiffness in healthy AT, and whether a change in material properties (i.e. Young's modulus) occurs in parallel, is still unclear.

This study aimed to investigate the acute effect of long-duration and high-intensity loading on the volume and stiffness of healthy free AT (i.e. pathology free or non-injured tendon). We hypothesized that a higher level of load duration and intensity could result in greater immediate reductions in tendon volume and stiffness compared with the effects of lower load duration and intensity.

## MATERIALS AND METHODS

### Trial design

We conducted a randomized crossover trial involving three different isometric interventions implemented over a period of 3 weeks and separated by a 1 week washout period between interventions, to ensure recovery of tendon properties (volume and stiffness) to baseline. A crossover design provides the advantage of controlling individual differences (confounders) as the subjects are their own controls, thus enabling more statistical power and a smaller sample size (Portney and Watkins, 2014). Reporting of this crossover trial is in accordance with the CONSORT statement for this trial type (Dwan et al., 2019) and the trial was prospectively registered (Australian New Zealand Clinical Trials Registry, http://www.anzctr.org.au; registration record: ACTRN12620000306910).

### Participants

Sixteen healthy volunteers (12 males, 4 females; age 24.4±9.4 years, body mass 70.9±16.1 kg, height 1.7±0.1 m) participated in this study. Participants were recruited from the local university student and staff population and via personal contacts of the researchers. All participants were recreationally active (see Table S1). Participants were excluded if they had current painful musculoskeletal disorders in the lower limbs, a history of lower limb surgery/trauma in the past 12 months (in the dominant side) or neurological conditions (i.e. multiple sclerosis, Parkinson's disease, stroke) or a history of AT pain or injury (in the dominant side). Ultrasound examination revealed no signs of AT disorders (i.e. thickening of the midportion and/or enthesis or hypoechoic regions) (Maffulli et al., 2003). The study was approved by Monash University Human Research Ethics Committee (Project ID: 21356). All participants provided written informed consent prior to participation.

### Interventions

Each participant attended the laboratory on three occasions, at the same time of day, with a 7 day interval between sessions. The participants were asked to refrain from strenuous physical activity 24 h prior to each testing session. One isometric intervention was performed per testing session in a random sequence (according to the allocated sequence for each participant). The isometric interventions varied in load duration (contraction duration) and load intensity but consisted of a similar volume (10 repetitions×4 sets) and a similar rest time between the repetitions (10 s) and sets (3 min) (Table 1). This allowed direct comparison amongst the interventions for the effects of different load duration and load intensity on the AT morphological and mechanical properties. To ensure that target load duration and load intensity for each intervention were consistently attained, a VBScript (Visual Basic Scripting) was created for each intervention using LabChart 8 software scripting (ADInstruments, Spechbach, Germany) and displayed on real-time visual feedback.

**Table 1.**
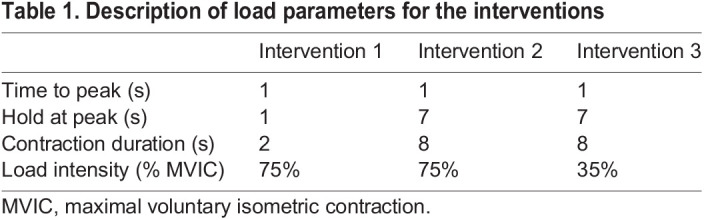
Description of load parameters for the interventions

**Fig. 1.**
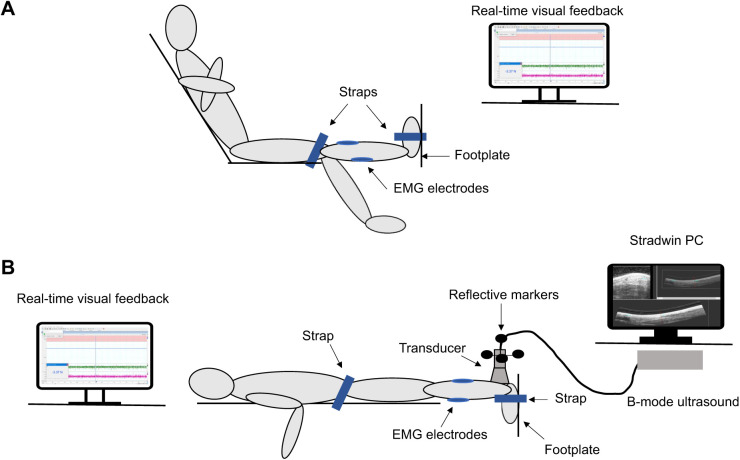
**Schematic diagram of the experimental setup.** (A) Setup during the interventions. (B) Setup during the maximal voluntary isometric contraction (MVIC) tests and freehand 3D scanning. The interventions were performed with the participant in a sitting position to avoid any discomfort associated with a prolonged prone position.

All interventions were undertaken with the participants seated in a custom-built dynamometer (Fig. 1A). The hip was in 120 deg of flexion, the knee was in full extension (knee angle 0 deg), and the tested ankle (dominant side) was in 0 deg plantar flexion (PF) with the lateral malleolus aligned to the axis of rotation of the dynamometer. The tested ankle was firmly strapped to the footplate of the dynamometer using a ladder strap to prevent downward foot slide during the intervention and was repositioned or tightened if movement was detected (this was done at the end of each set). To prevent knee flexion during PF contractions, a non-elastic strap was positioned above the knees. Participants were instructed to position both arms on the chest and to rest the non-tested leg down to the floor during the intervention to prevent compensation patterns.

In the first session and before commencing the isometric intervention, participants performed 3–5 trials of maximal PF isometric contractions (2–3 s) while seated in the dynamometer to establish target load intensity for the allocated intervention and subsequent interventions (i.e. 35% and 75%). Standardized instructions were provided during testing to motivate participants and ensure they achieved maximal PF torque. To prevent fatigue, participants were given 1–2 min rest between maximal PF isometric contraction tests and commencing the first intervention.

### Preconditioning and MVIC test

**Fig. 2.**
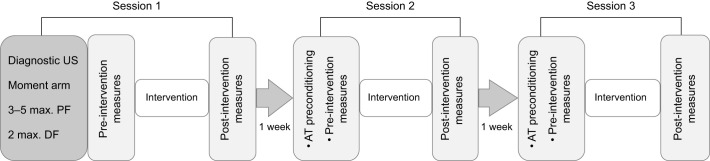
**Diagram of the testing procedure.** US, ultrasound; PF, plantar flexion; DF, dorsiflexion.

Tendon preconditioning was performed at each test session, followed by AT volume and stiffness assessment, the intervention, and then a repeat of the AT volume and stiffness assessment (Fig. 2). To precondition the tendon, participants performed five 80% MVICs of PF (3–5 s) whilst positioned prone on the same dynamometer (Fig. 1B). Both hips were secured to the dynamometer using a non-elastic strap to prevent forward body shift during PF trials and the ankle was kept in 0 deg plantar flexion and strapped to the footplate using a ladder strap. In the first session, employing identical positioning, tendon preconditioning was modified to include PF MVIC testing to establish torque levels for AT stiffness assessment (i.e. staged protocol; see below). The participants performed between 3 and 5 trials of ramped isometric PF contraction to a maximum, over 3–5 s duration. Maximal PF torque was confirmed to be attained when participants performed the two highest trials within 10% of each other. Standardized instructions were provided prior to testing (‘push toward the footplate and build up your force to maximum within 2–3 s, and hold at maximum for 2–3 s’) and during testing (‘push as hard as possible and hold for 2–3 s’) to motivate participants and ensure they achieved maximal PF torque. All torque data were displayed as real-time visual feedback, recorded at 1000 Hz with a PowerLab 26T, and analysed using LabChart 8 software (all ADInstruments).

### Freehand 3D ultrasound measures of AT volume and stiffness

Using freehand 3D ultrasound, the free AT volume and length were measured before and after each intervention (Fig. 2) whilst participants were positioned prone on the same dynamometer (Fig. 1B). The hips and knees were extended, and the tested ankle was in 0 deg plantar flexion with the lateral malleolus aligned to the axis of rotation of the force transducer. The freehand 3D ultrasound system that was utilized in this study has been described in previous studies investigating the mechanical and morphological properties of musculoskeletal tissues including the AT (Barber et al., 2009; Farris et al., 2013; Merza et al., 2021; Obst et al., 2014a) and the setup in the current study was identical to those we used in a previous study (Merza et al., 2021). The freehand 3D ultrasound includes a B-mode ultrasound device (ArtUS EXT-1H, Telemed, Vilnius, Lithuania) and a four-camera optical tracking system (OptiTrack PRIME 13, Tracking Tools v.1.7.1; NaturalPoint, Corvallis, OR, USA) that recorded the position and orientation of the transducer during scanning by tracking four reflective markers attached rigidly to the transducer (Treece et al., 2003). The acquired B-mode images were transformed into the global coordinate system using the Stradwin software package (v.5.4, Mechanical Engineering, Cambridge University, Cambridge, UK; http://mi.eng.cam.ac.uk/~rwp/stradwin) to construct a 3D image of the AT. Following the guidelines provided with the Stradwin software, temporal and spatial calibration of the ultrasound transducer was performed in a water bath (21°C) using a single-wall phantom calibration (Treece et al., 2003). After calibration, the coordinates of any pixel within a 2D ultrasound image were transformed into 3D space with an error of less than ±0.4 mm (Prager et al., 1998).

Freehand 3D ultrasound scanning was performed using a 40 mm linear transducer (L15-7 H40-A5, Telemed) with a central frequency of 7.5 MHz, a sampling frequency of 40 Hz, an axial and lateral resolution of 0.5 mm, and standardized image generation parameters (depth 40 mm, gain 50%, dynamic range 66 dB, power 0). To enhance visualization of the tendon cross-section and ensure the best contact between the transducer and skin, a disposable ultrasound gel pad 2×9 cm (Parker Laboratories, Fairfield, NJ, USA) was used during all scans. A thin layer of hypoallergenic ultrasound transmission gel (Other-Sonic, Pharmaceutical Innovations, Newark, NJ, USA) was applied to the participant's skin to reduce the friction between the skin and the standoff pad.

To obtain measurements of free AT morphology (i.e. resting length and cross-sectional area), two single-sweep 3D ultrasound scans were performed at rest (pre- and post-intervention). Scans were performed at a steady speed from calcaneal notch to soleus–muscle–tendon junction (MTJ). The duration of single scanning lasted between 15 and 20 s with approximately 0.1 mm distance between acquired ultrasound frames.

To estimate the free AT stiffness, two 3D ultrasound scans from calcaneal notch to soleus–MTJ were performed during sustained PF contractions at 35%, 55% and 75% MVIC in a random order (staged protocol). The PF torque levels for the staged protocol were standardized across the three testing sessions based on the values achieved in the first testing session. The participants were requested to hold each contraction until completion of scanning while maintaining the target force level, and were given 30–60 s rest between each trial to minimize fatigue. To minimize heel lift during the sustained submaximal PF contractions (and during the PF MVIC test), the ankle was firmly strapped to the footplate of the dynamometer using a ladder strap (Arya and Kulig, 2010). Infrequently, re-scanning was done in cases where the participant was unable to achieve or steadily maintain the target torque level or in the case of excessive heel lift (the strap was tightened prior to re-scanning). To reduce the influence of contraction duration (time under tension) on tendon viscoelastic behaviour and prevent tendon creep, an effort was made to standardize scanning duration at 8 s for all MVIC trials.

### Electromyography and muscle co-contraction

To account for the co-contraction of the tibialis anterior (TA) muscle during submaximal PF contractions and to obtain a true PF torque, maximal dorsiflexion (DF) contractions were performed (Arya and Kulig, 2010) (the position of the participant during the DF MVIC test was identical to that for the PF MVIC test – see above). The participants were asked to perform up to three trials of ramped maximal isometric DF contraction until the two highest were within 10% of each other. The participants were instructed to flex their foot against the ladder strap (i.e. isometric DF of the ankle) as hard as possible and hold for 2–3 s.

Bipolar surface electrodes (Kendall 300 Foam Electrodes, Covidien, Mansfield, MA, USA) were applied to the skin after shaving and cleansing with alcohol pads (see Hermens et al., 2000, for details). The raw EMG signals were recorded at 1000 Hz. Custom software (MATLAB R2019b, MathWorks, Natick, MA, USA) was used to process EMG data. A bandpass filter (20–450 Hz) was applied to eliminate electrical noise in the EMG data. The filtered signal was enveloped using the root-mean-square method over a sliding window of 200 samples. The antagonist DF torque was estimated from the relationship between TA EMG activity and recorded torque during DF contractions (Eqn 1), assuming a linear relationship between the recorded EMG amplitude and muscle torque (Kongsgaard et al., 2011). The estimated antagonist DF torque was added to the net PF torque to obtain a true estimate of the PF torque (Geremia et al., 2018; Kongsgaard et al., 2011; Arya and Kulig, 2010):
(1)

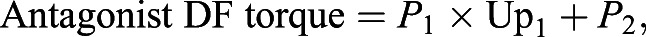
where *P*_1_ is the slope of the gradient of the relationship between TA EMG activity and recorded torque during the DF contractions, Up_1_ is the envelope of the TA EMG signal and *P*_2_ is the regression constant.

### Tendon moment arm

To obtain PF force, the resultant true PF torque was divided by the internal moment arm (Kongsgaard et al., 2011; Arya and Kulig, 2010). Moment arm was obtained at rest using B-mode ultrasound while the ankle joint was kept in 0 deg PF (Geremia et al., 2018). The inferior tip of the lateral malleolus of the tested ankle (i.e. centre of rotation, COR) was marked with a permanent marker. A 40 mm linear probe was placed longitudinally over the AT to acquire a static ultrasound image, with the centre of the probe aligned with the COR. The distance from the inferior tip of the ultrasound probe to the COR was measured using a tape measure (*d*_1_). From the B-mode static ultrasound image, the distance between the skin and the midline of the AT (*d*_2_), also known as the line of action (LOA) (Maganaris et al., 1998) was measured using the in-built length tool provided in the Echo wave II v.4.0.1 software. The difference between these two lines (*d*_1_*–d*_2_) represented the AT moment arm (i.e. the perpendicular distance from the COR to the LOA) (Geremia et al., 2018; Kongsgaard et al., 2011). The total mean and standard deviation of the moment arm for the 16 participants was 43.3±3.93 mm, which was comparable to values previously reported in the literature (Kongsgaard et al., 2011; Maganaris et al., 1998).

### Freehand 3D ultrasound image analysis and reconstruction of free AT volume

**Fig. 3.**
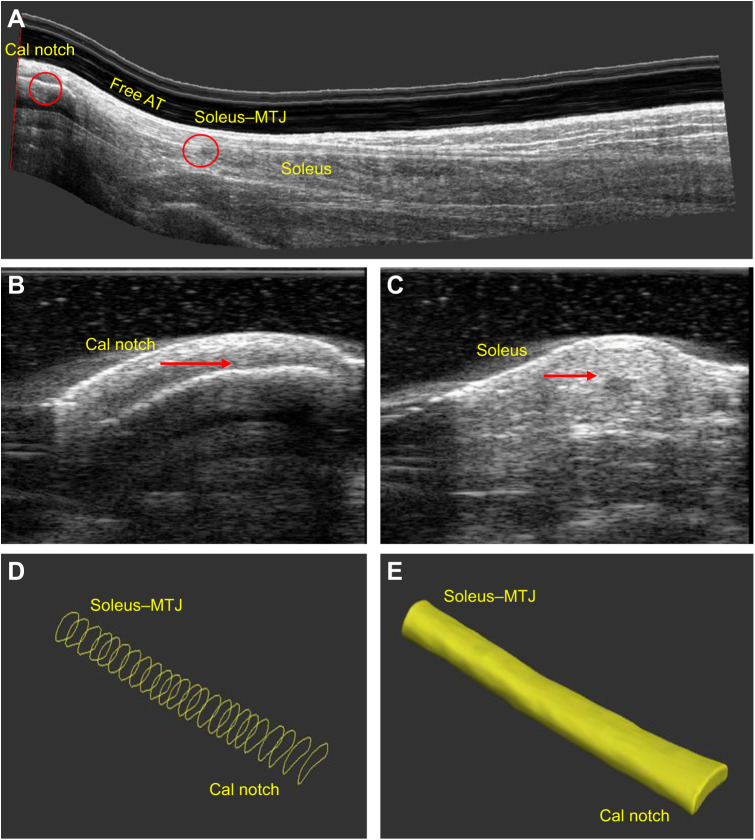
**3D Achilles tendon image analysis.** (A) Sagittal plane of a 3D ultrasound image of the free Achilles tendon (AT). Two anatomic landmarks [calcaneal notch and soleus–muscle–tendon junction (MTJ)] were identified on the (A) sagittal plane and (B,C) transverse plane for calculation of AT length. Cal notch, calcaneal notch. (D) Manual contouring of free AT cross-sections from calcaneal notch to soleus–MTJ. (E) Free AT volume reconstruction.

Stradwin software was used to perform image analysis. To measure free AT length (at rest and MVIC), two anatomical sites – the calcaneal notch and soleus–MTJ – were manually identified on sagittal and transverse images, then marked using a landmark tool (two-point method) (Fig. 3A–C). The soleus–MTJ was the first visible cross-section of muscle tissue and was determined using sagittal, frontal and transverse image planes. The distance between the calcaneal notch and soleus–MTJ was defined as the free AT and the length was measured using the length tool within the Stradwin software. Tendon cross-sections were manually contoured from the transverse B-mode images from the calcaneal notch to the soleus–MTJ at 5–10 mm intervals (Fig. 3D). The 3D tendon image reconstruction was performed on the segmented cross-sections using the in-built interpolation algorithm in the Stradwin software (Fig. 3E).

### Determination of free AT stiffness and Young's modulus

To calculate the free AT stiffness (N mm^−1^), all torque and EMG data obtained at maximal DF and submaximal PF trials, as well as data for the free AT length (i.e. at rest and during MVICs), were exported to MATLAB software R2019b (MathWorks). To measure the amount of tendon elongation at 35%, 55% and 75% MVIC, the mean resting length was subtracted from the mean length obtained at each corresponding MVIC level. The slope of the line fitted to the force–elongation curve (i.e. PF force at 35%, 55% and 75% MVIC and corresponding tendon elongation) across all force levels represented the individual stiffness. Tendon stiffness was normalized to CSA and length to determine Young's modulus, which was calculated by dividing tendon force by the average resting CSA (i.e. stress MPa) and tendon elongation by the resting length (i.e. strain %). The average CSA was determined from the contoured cross-sections using a custom MATLAB script. Young's modulus was the slope of the line fitted to the stress–strain curve across the entire stress range.

### Sample size calculation

The sample size was calculated using our pilot data (unpublished). We found a Cohen's standardized effect of ≥0.5 between the study interventions. Therefore, to achieve an effect size (ES) of ≥0.5 with 5% significance level and power of 80%, the sample size required for this crossover trial was *n*=11. This assumed a correlation between repeated measures of 0.2.

### Randomization

Each participant performed three different isometric interventions in a random sequence using a computer-based random sequence generation. An off-site researcher (P.M.) not involved in data collection informed the researcher who performed data collection (E.Y.M.) of the allocation sequence. This occurred immediately prior to the first session. Because of the nature of the interventions, the participants were aware of the exercise intervention they performed each week. However, we were very careful to ensure that the participants were not aware of the hypothesis of the study, and at no time was this disclosed in any written materials or verbal interactions.

### Reliability of tendon volume and stiffness measurements

Freehand 3D ultrasound has been shown to provide accurate measures of phantom volumes (±0.5 ml) and reliable measures for *in vivo* free AT volume (intra-class correlation coefficient (ICC) ≥0.98) (Obst et al., 2014a) and stiffness (ICC=0.994) (Merza et al., 2021). A recent study by Devaprakash et al. (2019) demonstrates high agreement between magnetic resonance imaging (MRI) and freehand 3D ultrasound estimates of free AT volume.

### Reliability of PF force

Between-session (using pre-intervention data) and within-session reliability of PF force at 35%, 55% and 75% MVIC was assessed using the intra-class correlation coefficient [ICC (3,1), two-way mixed-effects model with absolute agreement] and 95% confidence interval (CI), coefficient of variation (CV) and the minimal detectable change (MDC). The between-sessions and within-session ICCs for PF force at all MVIC levels were greater than 0.97 (lower band of 95% CI was ≥0.845). The CV and MDC ranged between 0% to 9% and between 68.5 to 179 N, respectively.

### Statistical methods

A (2×3) repeated measures ANOVA with time (pre–post intervention) and type of isometric intervention (1, 2 and 3) as within-subjects factors, was used to investigate the interaction and simple effects of the time and type of intervention on the following dependent variables: free AT volume and free AT stiffness. Data normality was evaluated with the Kolmogorov–Smirnov test. Effect size (ES) was reported for between-group differences (where ES is the difference between change scores/pooled change score s.d.) (Middel and Van Sonderen, 2002). All statistical analyses were performed using SPSS Statistics software (IBM SPSS Statistics for Windows, v.26.0, IBM Corp., Armonk, NY, USA). The level of significance for all the tests was set at *P*<0.05. Data are expressed as means±s.d.

## RESULTS

### Acute effect of isometric interventions on free AT volume

**Fig. 4.**
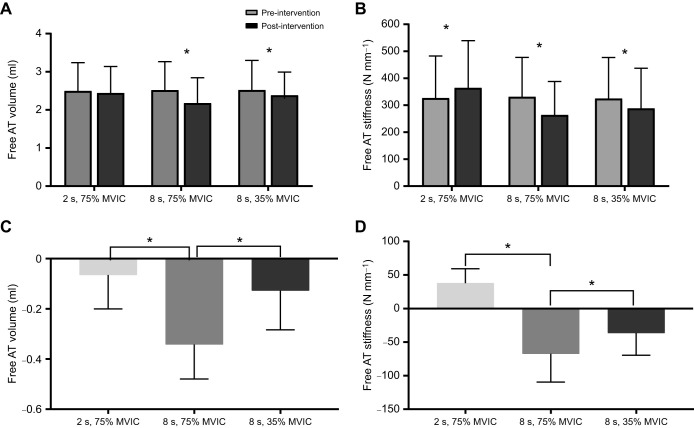
**Free AT volume and stiffness data.** (A,B) Pre- and post-intervention values of free AT volume (A) and stiffness (B). *Significant *post hoc* pairwise difference from baseline (pre-intervention; *P*<0.05, two-tailed). (C,D) Change score (post- to pre-intervention values) of the free AT volume (C) and stiffness (D) for all interventions. *Significant difference between interventions (*P*<0.05, two-tailed) according to interaction contrasts analysis. Data are means±s.d. (*n*=16).

There was a significant time-by-intervention interaction effect (*F*_2,30_=25.098, *P*≤0.001) on the free AT volume. Interaction (within-subjects) contrasts analysis shows that the acute effect of the 8 s, 75% MVIC intervention on the free AT volume was significantly greater than that of the 2 s, 75% MVIC (ES=2) and 8 s, 35% MVIC (ES=1.4) interventions but there was no significant difference between the 2 s, 75% MVIC and 8 s, 35% MVIC interventions (ES=0.5) on the free AT volume (Fig. 4C). The simple effects analysis shows that the 2 s, 75% MVIC intervention had no significant acute effect on the free AT volume (*P*=0.106), which changed only by an average of <−0.1 ml (2.3%), whilst the 8 s, 75% MVIC and 8 s, 35% MVIC interventions significantly reduced the free AT volume by an average of 0.3 ml (13.6%) (*P*≤0.001) and 0.1 ml (5.3%) (*P*=0.004), respectively (Fig. 4A). The same test indicates no significant differences in the free AT volume at baseline (pre-intervention) across interventions (*P*≥0.05). The 8 s, 75% MVIC intervention had the greatest acute effect on free AT volume. The pre- and post-intervention mean±s.d. free AT volume is shown in Table 2.

**Table 2.**
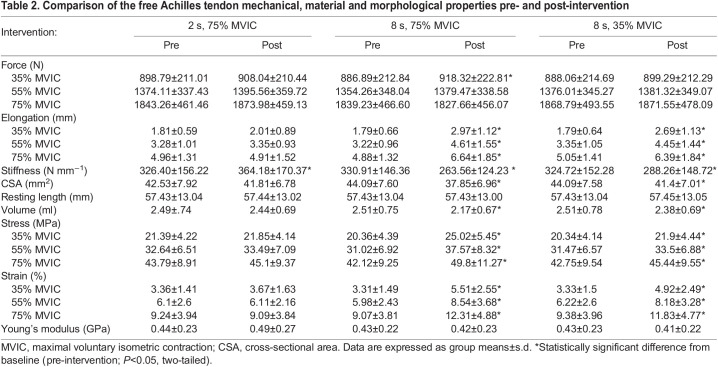
Comparison of the free Achilles tendon mechanical, material and morphological properties pre- and post-intervention

### Acute effect of isometric interventions on free AT stiffness

There was a significant time-by-intervention interaction effect (*F*_2,30_=60.503, *P*≤0.001) on the free AT stiffness. Interaction (within-subjects) contrasts analysis shows that the acute effect of the 8 s, 75% MVIC intervention on the free AT stiffness was significantly greater than that of the 2 s, 75% MVIC (ES=0.9) and 8 s, 35% MVIC (ES=0.8) interventions but there was no significant difference between the 2 s, 75% MVIC and 8 s, 35% MVIC interventions (ES=0.0) on the free AT stiffness (Fig. 4D). The simple effects analysis shows that the 2 s, 75% MVIC intervention significantly increased the free AT stiffness by an average of 37.78 N mm^−1^ (11.5%) (*P*≤0.001) and the 8 s, 75% MVIC and 8 s, 35% MVIC interventions significantly reduced the free AT stiffness by an average of 67.35 N mm^−1^ (20.4%) (*P*≤0.001) and 36.45 N mm^−1^ (11.2%) (*P*=0.001), respectively (Fig. 4B). The same test indicates no significant differences in the free AT stiffness at baseline (pre-intervention) across interventions (*P*≥0.05). The 8 s, 75% MVIC intervention had the greatest acute effect on free AT stiffness. The pre- and post-intervention mean±s.d. free AT stiffness, elongation and tendon force are shown in Table 2.

## DISCUSSION

The present study investigated the acute effect of long-duration and high-intensity loading on the morphological and mechanical properties of the free AT in healthy humans. Consistent with our hypothesis, the main finding was that a long-duration and high-intensity loading resulted in greater immediate reductions in the volume and stiffness of the free AT compared with lower levels of load duration and intensity.

### Acute effect of isometric interventions on free AT volume

The present study found that long-duration contraction at 75% MVIC reduced free AT volume by 60% more than long-duration contraction at 35% MVIC and by 82.3% more than short-duration contraction at 75% MVIC. The reduction in the free AT volume found in the current study is likely to be a manifestation of transient fluid flow and exudation. This is in light of the *ex vivo* investigations that demonstrated immediate fluid flow and reduced fluid volume as a response to cyclic and static load (Butler et al., 1997; Lavagnino et al., 2003; Hannafin and Arnoczky, 1994), and *in vivo* investigations using MRI (i.e. off-resonance saturation combined with ultrashort echo time sequence) that show reductions in AT volume and concomitant hydration state after cross-country running (Grosse et al., 2016; Syha et al., 2014). Fluid flow driven by the mechanical load might be attributed to increased hydrostatic pressure associated with the reorganization of collagen fibres (Connizzo et al., 2013; Atkinson et al., 1997). In contrast, the relatively constant tendon volume observed with short-duration contraction probably reflects a preserved fluid content.

The 13.5% reduction in AT volume following the long-duration contraction at 75% MVIC is higher than reductions reported following cross-country running (4% and 5.8%, respectively) (Syha et al., 2014; Grosse et al., 2016). This may be because running exposes the tendon to high mechanical stress but lasting only for a short duration (0.2–0.25 s) (Obst et al., 2013). Our findings suggest that the change in tendon volume occurs in a time-dependent manner (i.e. a greater volume reduction occurs with 8 s than with 2 s contraction), which may be attributed to the viscoelasticity of tendinous structures (Pearson et al., 2007). We also investigated the effect of long-duration contraction performed at a low load intensity (35% MVIC). Interestingly, we found a significant reduction in tendon volume by 5.4% (>0.1 ml, *P*=0.004) with long-duration contraction at 35% MVIC. The significant reduction in tendon volume following long-duration contractions was greater than our MDC (where MDC=standard error of measurement×1.96×√2) which was ±0.1 ml, and therefore was probably a real change, on average.

In contrast to our findings of reduced AT volume by 5.4% with 35% MVIC contractions, Nuri et al. (2017b) found that the AT volume of healthy humans, measured by freehand 3D ultrasound, remained constant after repeated isometric contractions at 50% MVIC (10 repetitions×25 s). Despite the comparable PF torque values between our study and that of Nuri et al. (2017b) (35 versus 40.5 N m, respectively), we had a higher number of loading cycles (i.e. 10 repetitions×4 sets) compared with Nuri et al. (2017b), meaning a longer total loading duration (i.e. 320 s versus 250 s). In this regard, several studies have shown that AT stiffness was significantly reduced following maximal isometric contractions (Kay and Blazevich, 2009, 2010; Kubo et al., 2002), and the study employing the longest total loading duration reported the greatest response (Kubo et al., 2002).

Our free AT volume (2.5 ml) is comparable to MRI data reported by Syha et al. (2014) and Grosse et al. (2016) (2.4 cm^3^ and 2.3 ml, respectively), and freehand 3D ultrasound data reported by Devaprakash et al. (2020) (<3 ml for trained runners and 3 ml for healthy controls). However, our free AT volume measurement is smaller than 3D ultrasound data reported by Obst et al. (2014b) and Nuri et al. (2017b) for healthy young males (which ranged between 3.9 and 4.9 ml). The average CSA value reported in the present study was comparable to 3D ultrasound data reported by Nuri et al. (2017b) (52 mm^2^) for the midportion of the free AT but smaller than MRI data reported by Devaprakash et al. (2020) (60 mm^2^).

### Comparison of volume and diameter changes

Previous *in vivo* studies investigated the acute effect of different loading interventions on tendon structural properties (i.e. thickness and diameter) and demonstrated immediate thickness/diametral reductions post-acute loading (Wearing et al., 2007; Grigg et al., 2012, 2009). For example, Wearing et al. (2007) reported a reduction of 15% in Achilles anterior–posterior diameter following resistive PF exercise and attributed such reduction to transient fluid loss. Importantly, the diametral reduction could probably occur with fluid redistribution within the tendon without the fluid leaving the tendon core (i.e. exudation). Therefore, it is unknown to what extent the reduction in tendon diameter reflects a change in tendon volume or fluid flow/exudation.

### Acute effects of isometric interventions on free AT stiffness

The present study found that long-duration contraction at 75% MVIC changed the free AT stiffness by 46% more than long-duration contraction at 35% MVIC and 44% more than short-duration contraction at 75% MVIC. While there were no changes in force across intervention conditions, there was a significant increase in corresponding tendon elongation following the interventions involving the long-duration contractions (8 s, 75% MVIC and 8 s, 35% MVIC) (Table 2). Therefore, the observed reduction in the free AT stiffness was also associated with an increased tendon elongation for a given force. Our stiffness value for the free AT (between 325 and 331 N mm^−1^) is comparable to the value reported by Devaprakash et al. (2020) (410±164 N mm^−1^) for the free AT, using 3D ultrasound.

Similar to our findings for volume, mechanical loading seems to influence the change in stiffness. We found that long-duration contraction at 35% and 75% MVIC reduced the free AT stiffness but the reduction at 35% was significantly smaller than that at 75% MVIC (11.3% versus 20.4%, respectively). In agreement with our finding, there is evidence that the stiffness of the vastus lateralis tendon was significantly reduced after long-duration contractions but not after repetitive drop jumps (Kubo et al., 2005) or shorter-duration (1 s) contractions (Kubo et al., 2001c). Here, we found reductions in tendon stiffness and concomitant volume immediately after long-duration loading with no changes in Young's modulus. This suggests that reductions in tendon volume through reductions in CSA (fluid flow/exudation) contribute to reductions in stiffness with acute loading. Theoretically, reduced fluid volume (i.e. reduction in the viscous element) would make the tendon thinner and may deprive the elastic matrix components of protection against tensile loading causing greater stress and strain (i.e. reduced stiffness). An unexpected finding was that the short-duration contraction at 75% MVIC induced a significant but small increase in the free AT stiffness by 11.5%. It is noteworthy that the changes in free AT stiffness following the 2 s, 75%, and 8 s, 35% interventions were smaller than our MDC value (≤45 N mm^−1^) and therefore within measurement error, on average. Notably, the between-group differences support our hypothesis of greater change in the intervention with the highest duration and intensity.

### Implications for tendon adaptation

Our findings may provide evidence that the applied load on the tendon must be heavy and sustained for a long duration to maximize immediate volume reduction. This acute tendon response may be important to shift the tendon toward optimal long-term adaptation; however, this remains speculative and requires further investigation.

Load-induced fluid flow/exudation may be a remodelling signal for tendon cells. This is likely to occur because radial fluid flow to the periphery (exudation) may temporarily reduce the fluid volume within the tendon core, enabling tenocytes to perceive loading. At the same time, the flow of tendon fluid generates shear stresses on the tenocyte membrane, all of which stimulate cellular activity (Wall et al., 2016; Docking et al., 2013; Ingber, 2006). Therefore, the immediate reduction in free AT volume observed after the long-duration high-intensity loading might be a physiological response that stimulates tenocyte mechanotransduction and signalling. We speculate that chronic exposure to long-duration load at high load intensity should lead to greater changes in tendon matrix and ultimately greater tendon adaptation (i.e. increased stiffness and tendon hypertrophy) compared with lower levels of load duration and intensity. In this context, Kubo et al. (2001a) found that tendon stiffness remarkably increased after 12 weeks of high-intensity isometric training with a longer-duration contraction versus a shorter duration.

### Limitations

The present study has some limitations that need to be considered. First, the level of PF force in the present study was not maximal (the highest force was exerted at 75% MVIC). The AT viscoelastic behaviour would probably differ under higher/maximal absolute tendon forces; that is, a higher force level would have caused the free AT to display greater stiffness (Rigby et al., 1959). However, with freehand 3D ultrasound scanning, sustaining a maximal contraction for long enough to attain high-quality images is extremely challenging. Second, we examined changes in volume and stiffness at the level of the free AT, which according to the evidence exhibits distinct material properties (i.e. higher longitudinal strain) compared with the proximal gastrocnemii tendon (Magnusson et al., 2003; Iwanuma et al., 2011). Therefore, our findings may not be generalizable to the gastrocnemius portion of the AT. Third, our analysis only presents volumetric changes across the entire free AT, and without conducting regional analysis, it is not possible to determine whether there are any region-specific changes in tendon volume. Fourth, although the direction of loading response for each intervention was the same in every subject, there was an inter-subject variability in the magnitude of change. Various levels of activity and plantar flexor strength amongst the participants may explain this variability. Future studies with specific inclusion criteria may be needed to examine whether there is a differential adaptive response between athletic and recreationally active populations and between men and women, separately. Finally, we had a small sample size of healthy participants (*n*=16) with a greater percentage of men (*n*=12) than women (*n*=4) (75% male). Therefore, we suggest caution in generalizing our results to other populations.

### Conclusion

The present study demonstrates that long-duration and high-intensity loading result in greater immediate reductions in the volume and stiffness of the free AT compared with lower levels of load duration and intensity.

## Supplementary Material

Supplementary information
