# Supplementary material for: The acute effects of higher versus lower load duration and intensity on morphological and mechanical properties of the healthy Achilles tendon: a randomized crossover trial
Source: J Exp Biol. 2022 May 13;225(10):jeb243741. doi: 10.1242/jeb.243741 (PMC9167578; doi:10.1242/jeb.243741)
Supplement: Supplementary information [file jexbio-225-243741-s1.pdf]

Table 1. Type of physical activity with corresponding hours per week among the participants.

Participant	Type of physical activity or exercise	Hours per week (number)
1	Football and basketball	5
2	Pilates and tap dancing	3
3	Basketball	2
4	Rock climbing	8
5	Gym classes and netball	4
6	Walking	3
7	Football, basketball, and gym	10
8	Basketball	2
9	Soccer, weightlifting, and basketball	6
10	Soccer and volleyball	10
11	Power lifting	8-10
12	Skating	30
13	Weightlifting	5
14	None	-
15	None	-
16	Swimming	2
